# Stretching of porous poly (l-lactide-co-ε-caprolactone) membranes regulates the differentiation of mesenchymal stem cells

**DOI:** 10.3389/fcell.2024.1303688

**Published:** 2024-01-25

**Authors:** Geonhui Lee, Seong-Beom Han, Soo Hyun Kim, Sangmoo Jeong, Dong-Hwee Kim

**Affiliations:** ^1^ Department of Chemical and Biomolecular Engineering, Johns Hopkins University, Baltimore, MD, United States; ^2^ Institute for NanoBioTechnology, Johns Hopkins University, Baltimore, MD, United States; ^3^ KU-KIST Graduate School of Converging Science and Technology, Korea University, Seoul, Republic of Korea; ^4^ Biomaterials Research Center, Biomedical Research Division, Korea Institute of Science and Technology, Seoul, Republic of Korea; ^5^ Department of Integrative Energy Engineering, College of Engineering, Korea University, Seoul, Republic of Korea

**Keywords:** mechanotransduction, PLCL, bioreactor, mesenchymal stem cell, differentiation, smooth muscle cell

## Abstract

**Background:** Among a variety of biomaterials supporting cell growth for therapeutic applications, poly (l-lactide-co-ε-caprolactone) (PLCL) has been considered as one of the most attractive scaffolds for tissue engineering owing to its superior mechanical strength, biocompatibility, and processibility. Although extensive studies have been conducted on the relationship between the microstructure of polymeric materials and their mechanical properties, the use of the fine-tuned morphology and mechanical strength of PLCL membranes in stem cell differentiation has not yet been studied.

**Methods:** PLCL membranes were crystallized in a combination of diverse solvent–nonsolvent mixtures, including methanol (MeOH), isopropanol (IPA), chloroform (CF), and distilled water (DW), with different solvent polarities. A PLCL membrane with high mechanical strength induced by limited pore formation was placed in a custom bioreactor mimicking the reproducible physiological microenvironment of the vascular system to promote the differentiation of mesenchymal stem cells (MSCs) into smooth muscle cells (SMCs).

**Results:** We developed a simple, cost-effective method for fabricating porosity-controlled PLCL membranes based on the crystallization of copolymer chains in a combination of solvents and non-solvents. We confirmed that an increase in the ratio of the non-solvent increased the chain aggregation of PLCL by slow evaporation, leading to improved mechanical properties of the PLCL membrane. Furthermore, we demonstrated that the cyclic stretching of PLCL membranes induced MSC differentiation into SMCs within 10 days of culture.

**Conclusion:** The combination of solvent and non-solvent casting for PLCL solidification can be used to fabricate mechanically durable polymer membranes for use as mechanosensitive scaffolds for stem cell differentiation.

## 1 Introduction

The development of biocompatible polymeric materials can provide functional substitutes for native tissues and is an attractive strategy for repairing damaged or diseased tissues and organs (Obradovic et al., 2010). Engineered tissues can serve as physiologically relevant models for quantitative *in vitro* studies of the biological mechanisms inherent in tissue development (Altman et al., 2002). Thus, developing a reliable bioreactor system that can control the extracellular environmental conditions to precisely regulate tissue-specific cellular responses is essential. Bioreactors designed to simulate physiological conditions have been utilized to assess the quality of engineered tissue models (Mun et al., 2013). Whereas previous bioreactors, such as spinner flasks and rotator wall vessels, offer improved cell culture efficiency compared to traditional culture dish systems (Sodian et al., 2002; Shaikh et al., 2010), recently developed perfusion bioreactors further prevent contamination of culture systems by constantly refreshing the culture media (Meng et al., 2014; Teixeira et al., 2014; Zhang et al., 2014). Thus, the long-term stability of the perfusion bioreactor enables stem cell culture through additional preparation optimization.

To regulate the proliferation and differentiation of stem cells more accurately, controlling cellular physiology is important. Several studies have demonstrated that mechanical stress applied to stem cells enhances tissue regeneration because stem cell maintenance and differentiation are governed by a unique local microenvironment (Watt and Hogan, 2000; Even-Ram et al., 2006) and mechanical stimuli, and the physical setting of the external environment can directly alter cellular phenotype and functionality (Maul, Chew, Nieponice, Vorp, and mechanobiology, 2011). The bone marrow is one of the most promising sources of regenerative cells (Winer et al., 2008), and bone marrow-derived mesenchymal stem cells (BMSCs) with proliferative capabilities can be differentiated to form bones, cartilages, chondrocytes, adipocytes, and myoblasts (Friedenstein et al., 1970; Krampera et al., 2006; Chamberlain et al., 2007). As the differentiation potential of BMSCs is highly sensitive to changes in their physical environment, one of the primary goals of mechanobiological tissue engineering is to accelerate their differentiation into mature cells, ultimately enhancing the mechanical and regenerative properties of engineered tissues. For example, uniaxial cyclic stretch can promote the differentiation of BMSCs toward smooth muscle cells (SMCs) (Maul et al., 2011).

The effects of external forces, including mechanical strain, compression, and fluid shear stress, on the cellular function have long been studied not only in tissue engineering but in cardiovascular tissues, skeletal muscles, and adult stem cells (Osol, 1995; Wang and Thampatty, 2008; Sun et al., 2012). The physical conditions of an extracellular matrix, such as topography, roughness, porosity, and stiffness, are particularly important for determining the mechanical settings to which cells respond. For instance, the surface roughness and elastic modulus of a polymer membrane have been extensively studied to alter cellular responses such as proliferation and differentiation across cell types (Engler et al., 2006; Kunzler et al., 2007), without modifying its chemical composition. Moreover, because the mechanical compliance of tissue scaffolds determines the sensitivity of stem cells to tissue-level elasticity (Discher et al., 2005; Shin et al., 2008), controlling the mechanical properties of polymeric materials by substituting for the *in vivo* tissue matrix is a promising strategy for guiding the differentiation of BMSCs along neuronal, muscle, or bone lineages (Chowdhury et al., 2010).

Poly (l-lactide-co-ε-caprolactone) (PLCL) is an attractive polymeric material for use in tissue substitutes because of its mechanical durability as well as biocompatibility. (Shin et al., 2008; Mun et al., 2012; Mun et al., 2013). Recent studies have shown that biodegradable polymer films, including PLCL, poly (l-lactic acid) (PLLA), and poly (glycolic acid) (PGA), have been developed by *solvent-casting* technique (Sachlos and Czernuszka, 2003), where controlling the phase separation rate can determine the microstructure of the membrane (Diban and Stamatialis, 2011), which further differs in stem cell differentiation (Richardson et al., 2006).

Herein, we demonstrate a cost-effective and robust method for the fabrication of PLCL membranes via the crystallization of PLCL copolymer chains in a combination of solvents and non-solvents. The crystallization behavior of the PLCL copolymer was driven by the dissolution of the solute in the solvents, enabling changes in the porosity of the PLCL membranes. We showed that the degree of crystallization of the solvent-nonsolvent mixture determines the mechanical properties of PLCL membranes. Using our mechanically stable PLCL membranes to induce physical stimuli in BMSCs, we demonstrated that cyclic stretching could differentiate BMSCs into smooth muscle cells (SMCs) in a custom-made microfluidic system.

## 2 Materials and methods

### 2.1 PLCL matrix preparation

The co-polymerization of PLCL was performed as described previously (Kim S. H. et al., 2015). Briefly, PLCL (50:50) was polymerized in a 100-mL glass ampule containing L-lactide (100 mmol) and e-caprolactone (100 mmol) at 150°C for 24 h in the presence of 1,6-hexanediol (0.5 mmol) and stannous octoate (1 mmol) as a catalyst. The ampule was sealed under vacuum after being purged three times with nitrogen at 90°C and heated to 170°C for 24 h with stirring in an oil bath. After the reaction, the obtained polymer was dissolved in chloroform and filtered through a 4.5 mm pore membrane filter. The polymer was precipitated in excess methanol, filtered, and dried under vacuum. Initially, PLCL was well-dispersed in chloroform (CF). A 1-mL PLCL solution was injected into a 20 mL coagulation bath that consisted of six types: methanol, methanol/CF (3:1), methanol/distilled water (DW) (3:1), isopropanol (IPA), IPA/DW (3:1), and IPA/CF (3:1). The solid structure of PLCL could be distinguished by its white color as the polymer chain aggregation saturated. The resulting product was washed with methanol to dehydrate residual water.

### 2.2 Microchip fabrication

Microfluidic chips were fabricated using an SU-8 photoresist-patterned silicon wafer as a mold, as previously described (Lee G. et al., 2017; Lee G. H. et al., 2017). Briefly, the polydimethylsiloxane (PDMS) pre-polymer and cross-linker (Sylgard 184, Dow Corning) were mixed in a 10:1 ratio, poured onto a mold, and cured at 80°C for 2 h. The PDMS substrates and PLCL matrix were then bonded together using oxygen plasma treatment. The device consists of two microchambers separated by a flexible PLCL matrix. The microfluidic chip consisted of three layers: i) cell culture chamber (upper layer), ii) flexible PLCL matrix for transmitting stretch, and iii) actuation chamber for controlling the stretch. The prepared devices were heated in an oven at 80°C for 1 day to recover their hydrophobic surface properties.

### 2.3 Cell culture

BMSCs were purchased from ATCC and cultured in a medium containing alpha-MEM (Gibco-BRL-Invitrogen), supplemented with penicillin (100 U/mL), streptomycin (100 μg/mL), 1 mM sodium pyruvate (Gibco-BRL, CA, United States), and 10% FBS (Gibco). BMSCs were cultured in a humidified atmosphere containing 5% CO_2_ and 95% air at 37°C. BMSCs, passage seven to nine, were seeded on the fibronectin-coated (10 μg/mL) PLCL matrix at a concentration of 2,500 cells per matrix in a volume of 100 μL for differentiation and 5,000 cells per matrix in a volume of 100 μL for proliferation and incubated overnight to allow attachment to the PLCL substrate. The cell culture medium in the chamber was refreshed every day for 10 days. Strain was applied gradually on the PLCL matrix to allow the cells to orient: no strain on days 1 and 2, 3% strain on day 3; 5% strain on day 4; 7% strain on day 5; 10% strain on day 6; and 15% strain on days 7–10. For the SMC positive control, BMSCs were cultured with the differentiation media (alpha-MEM with 5 ng/mL TGFβ-1) for 8 days.

### 2.4 Dynamic bioreactor system

The bioreactor was designed as a closed-loop perfusion system providing a mechanical environment for BMSC culture under dynamic conditions. The bioreactor consists of three parts: a custom-designed push/pull syringe pump (control box), microfluidic cell culture chamber, and osmotic pump. A custom-made bioreactor system was developed to deliver constant physical stimulation by controlling the reciprocating motion of a 1 mL syringe. The bioreactor comprises a linear servo actuator (PoteNit, LSA-3024SD), I/O control button, and microcontroller (Atmel, Atmega128). A pressure sensor (Autonics, PSB-01C) was used to measure pressure on the PLCL membrane. Pressure fluctuation data were acquired in real time using custom-programmed LabVIEW with a DAQ board (USB-6009). Steady or pulsatile flow was generated by the bioreactor over the range of 0.5–2.6 uL/min to apply 3%–15% strain on the PLCL membrane in the fluidic chip.

### 2.5 Characterization of the PLCL matrix

The morphologies of the matrix considering non-solubility were analyzed using scanning electron microscopy (SEM; Hitachi). The mechanical properties according to different non-solvent compositions were analyzed using a unidirectional tensile machine (UTM).

### 2.6 Cell proliferation assay

The cell counting kit (CCK) assay (Dojindo) was used to investigate the adherence and proliferation of BMSCs on the PLCL matrix. The cells were seeded on the PLCL matrix without stimulation for 2 days and incubated for 8 days under shear stress or stretching. Then, 10 μL of CCK solution was added to each well, followed by 1 h of incubation. Its optical density was measured using a plate reader (Synergy H4 Microplate Reader, Biotek).

### 2.7 Immunostaining

The PLCL matrices were fixed in 4% paraformaldehyde (Sigma-Aldrich) for 1 h. After rinsing in phosphate-buffered saline (PBS), the samples were immersed in 1% Triton X-100 (Sigma-Aldrich) prior to permeabilization for 20 min, followed by washing with 0.1% bovine serum albumin/phosphate buffered saline solution (BSA/PBS). The samples were blocked for 30 min at room temperature with 3% BSA/PBS and washed again with 0.1% BSA/PBS. Subsequently, the samples were incubated in a 1:200 dilution of rabbit anti-α-smooth muscle actin overnight at 4°C. After another series of washes in 0.1% BSA/PBS, the cells were incubated for 2 h with a 1:1000 dilution of Alexa Fluor 599. The samples were stained with DAPI and observed under a fluorescence microscope (Olympus, Tokyo, Japan).

### 2.8 Shear stress calculation

The velocity field was calculated using computational fluid dynamics simulations on COMSOL. The fluid flow is described by the incompressible Navier-Stokes equation:

$$\rho \frac{\partial u}{\partial t}-\nabla \cdot \mu \left(\nabla u+{\left(\nabla u\right)}^{T}\right)+\mu u\cdot \nabla u+\nabla p=0$$

∇⋅u=0

where ρ is density, u is velocity, μ is viscosity, and p is pressure.

### 2.9 Cell alignment analysis

To quantify cell alignment, fluorescence images of actin-labelled cells stained with phalloidin were taken from three biological replicates for each condition and analyzed using the Orientation J plugin in the ImageJ software. The degree of cell orientation was determined by comparison with the longitudinal axis of the nucleus. More than 50 cells were analyzed per condition.

### 2.10 Porosity measurement

The PLCL membrane was cut into 1 × 1 cm pieces for high-resolution imaging using a scanning electron microscope (JSM-6701F, JEOL Ltd.). The images were then imported into the ImageJ software to determine the porosity of the PLCL membranes, which was quantified by analyzing the void spaces and pores present in the region of interest by adjusting the threshold.

### 2.11 RNA extraction and quantitative polymerase chain reaction (qPCR) analysis

To assess the differentiation of mesenchymal stem cells (MSCs) into smooth muscle cells (SMCs), the cells were collected and their RNA was isolated using an RNeasy Kit (Qiagen) in accordance with the manufacturer’s guidelines. Subsequently, quantitative polymerase chain reaction (qPCR) was performed utilizing iTaq-SYBR Green (Bio-Rad) and primers (Integrated DNA Technologies) with the CFX 384 Touch Real-Time PCR detection system (Bio-Rad). The following primers were used: ACTA2 (F: CCAGAGCCATTGTCACACAC/R: CAG​CCA​AGC​ACT​GTC​AGG), CNN1 (F: GCATGTCCTCTGCTCACTTCAA/R: GGG​CCA​GCT​TGT​TCT​TAA​CCT), and GAPDH (F: GCACCGTCAAGGCTGAGAAC/R: TGG​TGA​AGA​CGC​CAG​TGG​A). GAPDH mRNA levels served as a reference for normalizing the qPCR data.

### 2.12 Statistical analysis

All statistical analyses were performed using the GraphPad Prism software (GraphPad Software). Significance was assessed using the Student’s t-test for comparing two groups and one-way ANOVA for comparing more than two groups with an assumed Gaussian distribution and equal variances. All experiments were independently repeated at least three times, unless otherwise specified.

## 3 Results

### 3.1 Development of microfluidic device for MSC differentiation on PLCL membrane

**FIGURE 1:**
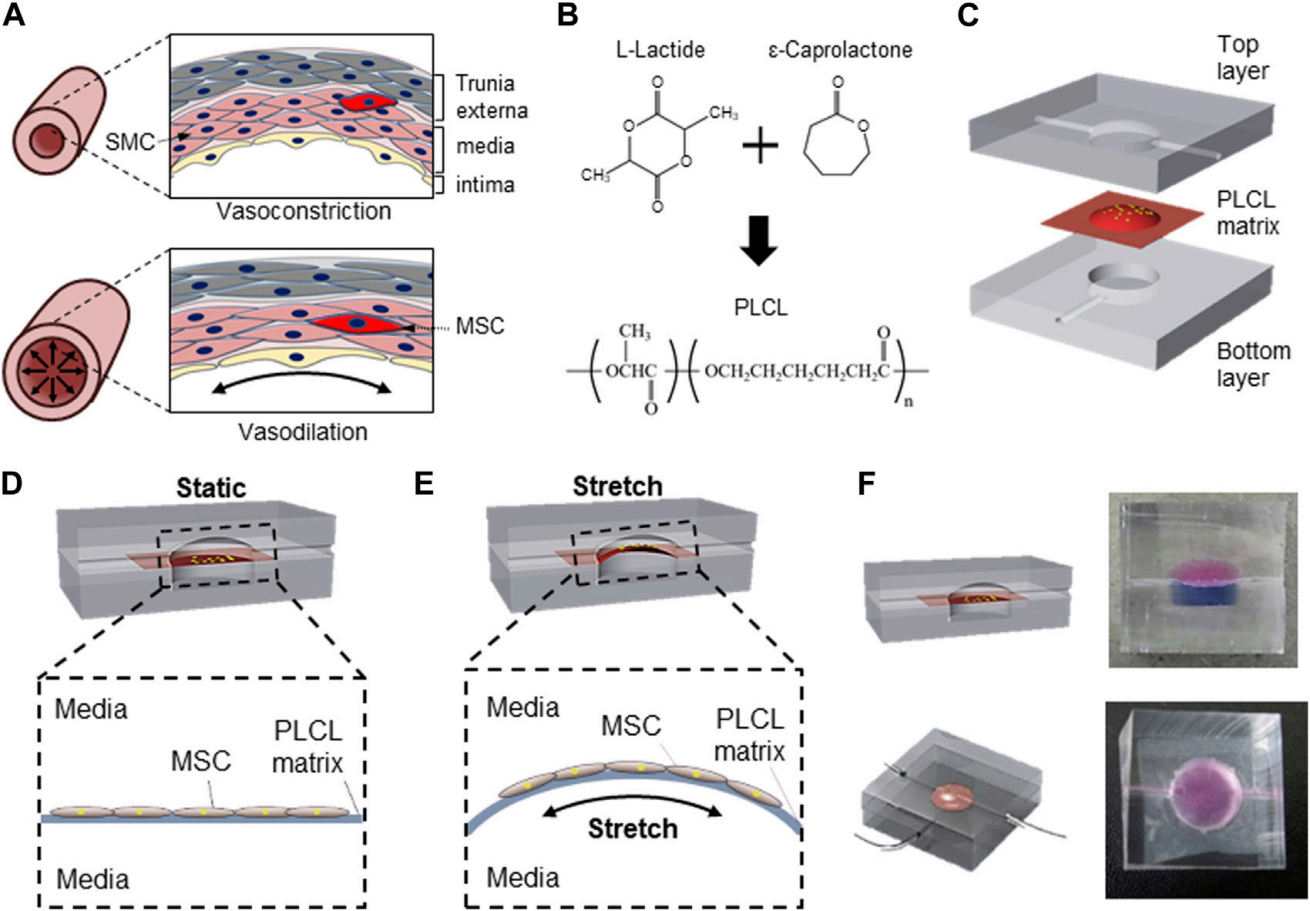
Schematic of PLCL membrane stretching system to mimic *in vivo* mesenchymal stem cells differentiation into smooth muscle cell. **(A)** Schematic of vascular structure during vasoconstriction and vasodilation. **(B)** Schematic diagram of PLCL copolymer synthesis. **(C)** Microfluidic device comprising three components including top layer for application of interstitial flow, interlayer of PLCL membrane for cell-adherent deformable substrate, and bottom layer for membrane stretching. Schematics of static **(D)** and stretched **(E)** PLCL membranes culturing MSCs in the microfluidic device. **(F)** Images of sectional and top views of a designed microfluidic device, where the top and bottom chambers are visualized with red and blue dyes, respectively.

Vascular support is critical for the survival and functional maintenance of cells and tissues. MSCs, one of the major seed cells for tissue engineering (Tuan et al., 2002), differentiate into SMCs to recover from tissue injury (Figure 1A). Because mechanical stimuli accelerates the proliferation of diverse stem cells (Delaine-Smith and Reilly, 2012; Potter et al., 2014; Sun et al., 2022), and continuous contraction and relaxation of muscular wall in the blood vessel represent extracellular physical cues (Webb, 2003), we synthesized a highly flexible PLCL copolymer membrane (Figure 1B). To further mimic the unique cyclic stress during vasoconstriction and vasodilation, we designed a novel microfluidic device comprising three layers: i) a top PDMS layer to mimic the interstitial flow to the cell, ii) synthesized PLCL membrane for the highly stretchable cell culture substrate, and iii) bottom PDMS layer to stretch the deformable membrane under cyclic fluidic pressure (Figure 1C).

The separation of the top and bottom layers by the PLCL membrane enabled the switch of cells between mechanically unstressed static conditions (Figure 1D) and mechanically stressed membrane-stretched conditions (Figure 1E) by controlling the fluid pressure in the bottom layer. The maintenance of complete separation between the upper and lower chambers with a PLCL membrane was confirmed by differently colored dyes (Figure 1F), and switching between static and stretch conditions by changing the fluid pressure in the bottom layer did not result in the mixing of colored dyes in each chamber. Moreover, because the top chamber is connected to the media reservoir and an osmotic pump to mimic interstitial flow, our newly designed device enables the simultaneous application of membrane stretching and interstitial fluid to MSCs cultured on PLCL membranes.

A computational simulation of the fluid shear stress further confirmed that the strain generated the highest shear stress, whereas the flow mimicking the interstitial flow barely generated shear stress in our microfluidic device system (Fig. S1).

### 3.2 Characterization of bioreactor for stretching system

**FIGURE 2:**
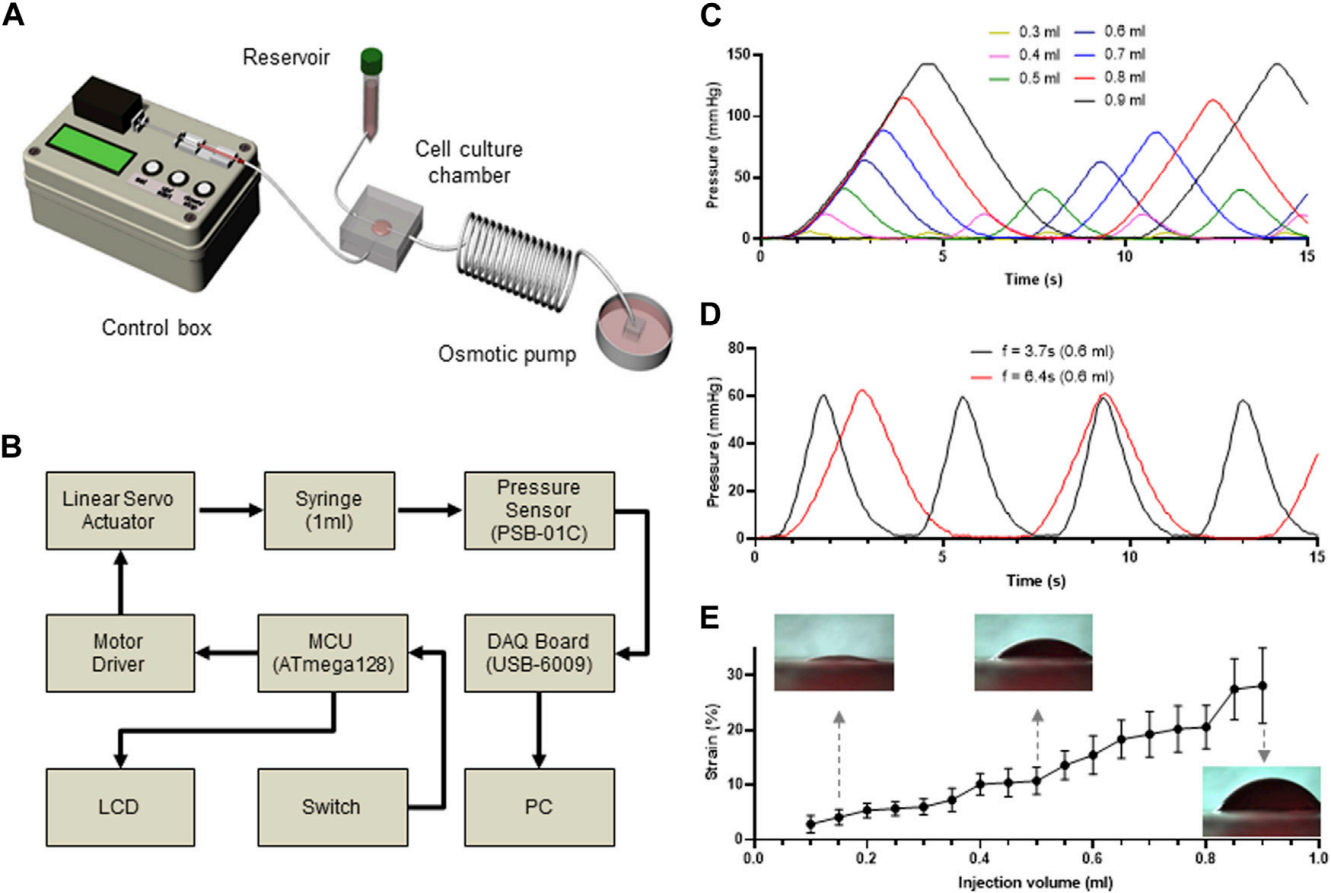
Custom-made microfluidic system to apply mechanical stimuli to the cultured cells. **(A)** Schematic view of a custom-made bioreactor to mimic the physiological stimuli. The microfluidic device is connected to a syringe pump to stretch the membrane and a media reservoir to mimic interstitial flow. **(B)** Workflow of controlling system to regulate membrane stretch and pressure measurement in the microfluidic device. **(C–D)** Time-lapse monitoring of membrane stretching. Differential pressure changes in the bottom layer of the microfluidic device indicate that membrane stretch is precisely controlled by changing injected fluid volumes **(C)** and injection frequency **(D)**. **(E)** Injected fluid volume dependent strain rate of the PLCL membrane.

To further develop a cost-effective and miniaturized bioreactor to promote the differentiation of MSCs into SMCs in a physiological microenvironment-mimicking microfluidic device, we connected the PLCL membrane-containing microfluidic device to the media reservoir and outlet, enabling long-term cell culture without manually changing the media, and a conventional 1 mL syringe mounted on a control box, enabling cyclic stretching of the PLCL membrane (Figure 2A). Because the reciprocating movement of the mounted syringe generated cyclic stretching of the PLCL membrane by varying the syringe-induced fluid pressure, we connected the bottom layer to a pressure sensor to measure the fluid pressure in the bottom chamber, which was fully monitored using a DAQ board (Figure 2B and Supplementary Figure S2).

As expected, the pressure changed proportionally with the injected fluid volume (Figure 2C), which was further confirmed by performing the same experiments at different frequencies of cyclic movement, where the peak-to-peak distances were constant for each frequency (Figure 2D). Therefore, by capturing the side view of the PLCL membranes that changed in response to differential changes in fluid pressure, we showed that the strain level was proportional to the injected volume in the bottom chamber (Figure 2E and Supplementary Video S1). These results indicate that our custom-made bioreactor system with an integrated PLCL membrane-embedded microfluidic device can precisely control the application of mechanical stimuli to membrane-adherent cells.

### 3.3 Solvent-dependent crystallization of PLCL chain

The mechanical properties of diverse polymeric materials can be tuned precisely by modifying their microstructures (Yan et al., 2016). Whereas crystallization is induced by the difference in the melting temperature (T_m_) of polymer components, leading to imperfect crystallization of each unit block (Sarasua et al., 1998), has been considered a robust and cost-effective method to improve the mechanical properties of copolymers (He and Xu, 2012), the crystallization of certain copolymers, such as PLCL, occurs simultaneously at a similar T_m_ (Fernández et al., 2012). Accordingly, the crystallization-dependent morphological alteration of the polymer membrane can be controlled by other factors such as the solvent, molecular weight, and polymer ratio (Saha et al., 2006). In this study, we tested whether pairing a solvent and non-solvent could alter the microstructure of PLCL membranes that could be utilized in our mechanical stimuli-induced stem cell differentiation bioreactor system.

**FIGURE 3:**
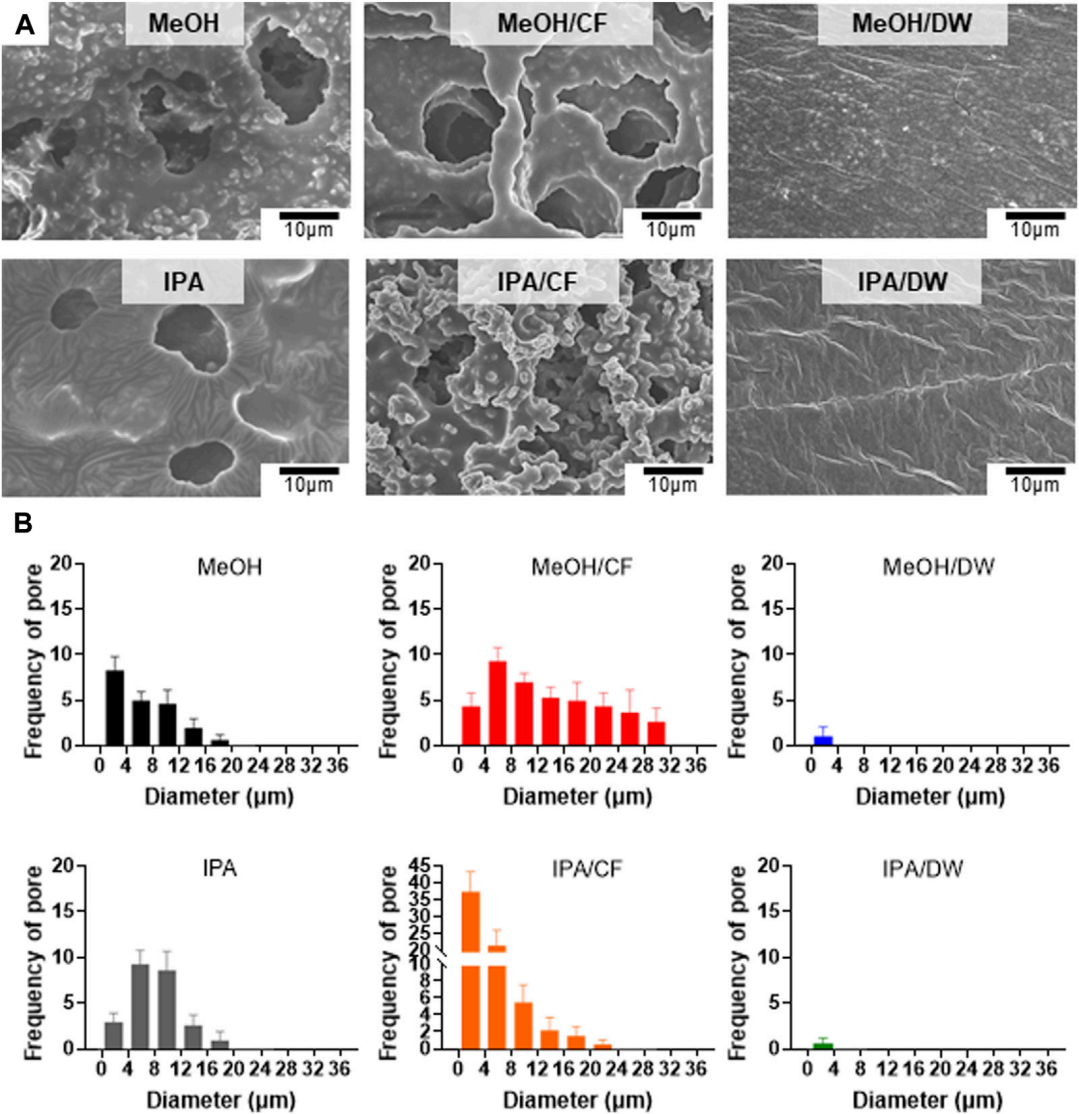
Crystallization-dependent differential microstructure of PLCL membranes. **(A)** Representative SEM micrographs of solidified PLCL membranes using methanol (MeOH), MeOH/chloroform (CF), MeOH/distilled water (DW), isopropanol (IPA), IPA/CF, and IPA/DW. **(B)** Distribution of pores in PLCL membranes crystallized by diverse non-solvent conditions. The pore is minimally detected in PLCL membranes solidified by DW-mixed solvents (MeOH/DW and IPA/DW).

We used methanol (MeOH) and isopropanol (IPA) and their pairs of chloroform (CF) and distilled water (DW) as non-solvent mixtures to crystallize the PLCL membranes (Figure 3). Top-down SEM images showed that the addition of CF led to a more porous microstructure than the PLCL membranes prepared using MeOH or IPA only, but mixing with DW largely suppressed the formation of the porous microstructure (Figure 3A). Because IPA has a solvent polarity index similar to that of MeOH (Razali et al., 2017) and the pore formation of the copolymer depends on the evaporation speed of the solvent (Phillip et al., 2010), the slowest evaporation of DW among the non-solvents could allow the complete phase separation of PLCL copolymers from the solvent mixture. The microstructure and morphology of copolymers such as separated phases and porosity are determined by the solvent evaporation and the lowest evaporation rate of DW among the nonsolvent could induce clear phase separation of PLCL from the original solvent. To determine if aggregated microstructures of the PLCL matrix are formed by the differential crystallization in response to diverse combinations of solvent and nonsolvent, we applied differential scanning calorimetry, where we noted that the PLCL copolymers exhibited a T_m_ (Supplementary Figure S3), which confirmed the formation of semi-crystalline structure of the PLCL copolymers. Thus, consistent with previous data (Figure 3A), the quantification of pores formed in the solvent-specific PLCL membranes revealed that adding DW to MeOH or IPA significantly reduced the number and size of pores, whereas CF inversely affected the formation of pores (Figure 3B).

These results imply that varying the combination of solvent and non-solvent can affect the mechanical properties of PLCL membranes by altering their microstructures.

### 3.4 Mechanical characterization of PLCL membrane different solvents

**FIGURE 4:**
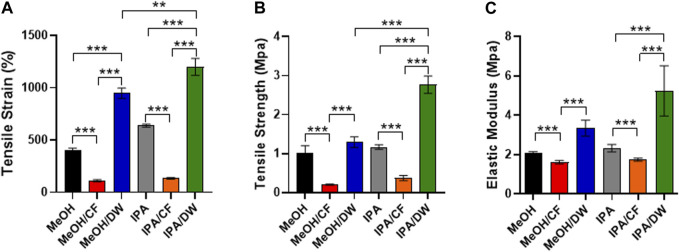
Characterization of mechanical properties of PLCL membranes prepared by different solvents. Tensile strain **(A)**, tensile strength **(B)**, and elastic modulus **(C)** of PLCL membranes crystallized by diverse non-solvents and their mixture. PLCL membranes solidified by DW-mixed solvents (MeOH/DW and IPA/DW) show relatively enhanced mechanical properties and the highest tensile strain and strength are detected in PLCL membranes crystallized by IPA/DW (green). Error bars indicated S.E.M., 1-way ANOVA using Tukey’s test was applied (**: *p* < 0.005; ***: *p* < 0.001; n = 3).

Because stem cell differentiation is highly mechanosensitive (Rammensee et al., 2017) and the formation of pores can alter the mechanical properties of membranes (Oh et al., 2010), we measured the tensile strain and tensile strength of the PLCL membranes prepared using different solvent-nonsolvent pairs using a universal tensile testing machine (Figure 4). The PLCL membrane with lower porosity, that is, the one crystallized by MeOH/DW or IPA/DW, exhibited higher tensile strain and tensile strength than the porous membrane prepared by MeOH or IPA alone. The addition of CF significantly reduced the mechanical strength (Figures 4A–C). Specifically, MeOH/CF induced the lowest elongation at the breaking point (111% ± 9.0%) and tensile strength (0.20 ± 0.01 MPa), and the highest elongation at the breaking point matrix (1200% ± 65.9%) and tensile strength (2.76 ± 0.18 MPa) in the IPA/DW (Figures 4A, B).

These results indicate that the mechanical properties of PLCL membranes can be determined by varying the combination of solvents and non-solvents during the crystallization process. Moreover, wide angle X-ray diffraction analysis (WAXD) showed that all PLCL membranes displayed similar primary peak levels (2θ = 19.4°) (Supplementary Figure S4). This result further indicated that the PLCL membranes maintained a similar degree of crystallinity regardless of the solvent conditions. These results suggest that the PLCL membranes crystallized using IPA/DW are the most durable polymeric materials for use in bioreactors aimed at mechanical stimuli-induced stem cell differentiation.

### 3.5 Mechanical stretch induced BMSC differentiation into smooth muscle cells

BMSCs can differentiate into SMCs in response to cyclic stretching (Choi et al., 2007; Ghazanfari, Tafazzoli-Shadpour, Shokrgozar, and communications, 2009). To assess the feasibility of stem cell differentiation using our stretchable PLCL membrane-equipped bioreactor system, we applied three different physical cues to the BMSCs cultured in our microfluidic device: i) no cues, ii) interstitial flow, and iii) strain, specifically mimicking the *in vivo* physiological conditions. Interstitial flow was enabled by an osmotic pump connected to the upper chamber (Figure 2A) (J. Y. Park et al., 2007), and matrix strain was induced using the most durable PLCL membrane crystallized from the IPA/DW solvent mixture, where the fluid pressure applied to the bottom layer of the microfluidic device induced cell strain (Figures 1, 2).

**FIGURE 5:**
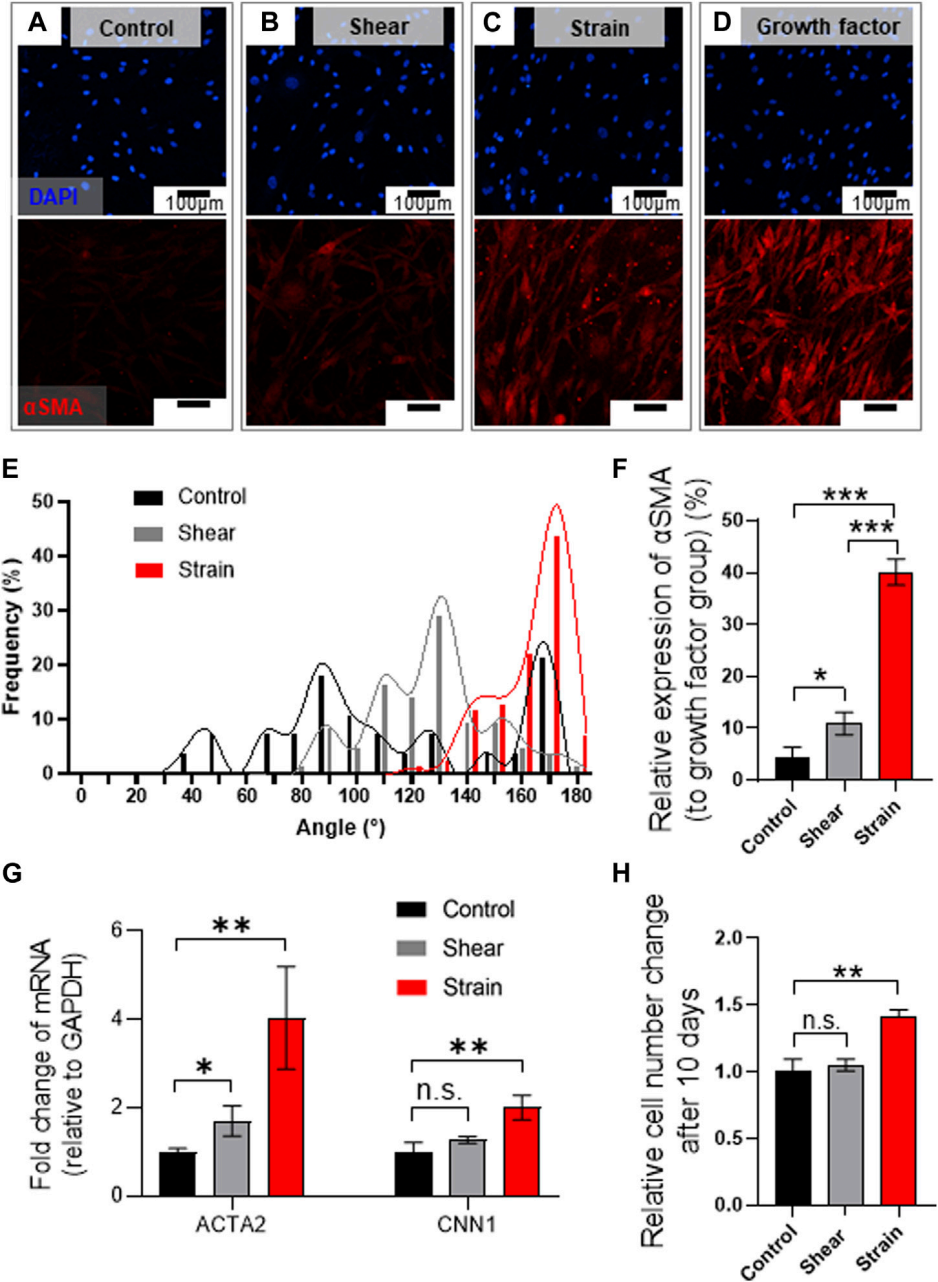
Mechanical stretch induces BMSC alignment and differentiation into SMCs on PLCL membrane. **(A–E)** Mechanical stress-induced morphological alteration. Immuno-stained BMSCs marking cell nucleus (DAPI, blue) and αSMA (red) were cultured on the PLCL membrane without mechanical stress (control, **(A)**, and with interstitial flow **(B)** or strain **(C)** or growth factor **(D)** for 8 days. Assessment of mechanical stimuli-dependent differentiation of BMSC indicates that strain induces highly aligned cell morphology **(E)**. **(F–H)** Quantitative analysis of mechanical stimuli-induced cell differentiation in PLCL membranes solidified by DW-mixed solvent (IPA/DW). Quantitative analysis of immunofluorescence microscopy **(F)** and qPCR **(G)** indicate higher differentiation into smooth muscle cells in the strain group. Stretch-induced enhanced proliferation of SMCs were determined by the CCK assay after 10 days of the experiment **(H)**. Error bars indicated S.E.M., 1-way ANOVA using Tukey’s test was applied (NS: not significant; *: *p* < 0.01; **: *p* < 0.005; ***: *p* < 0.001).

Immunofluorescence analysis marking α-smooth muscle actin (αSMA) implies a mechanical stimuli-dependent differential differentiation of BSMC to SMC (Figures 5A–C), where we noted the strain condition was most effective route for the differentiation (Figure 5C) compared to positive control differentiated by growth factor (Figure 5D). We observed that BMSCs cultured in PLCL membranes were highly aligned, which is consistent with SMCs *in vivo*, showing cell alignment along the direction of muscle contraction (Huycke et al., 2019), whereas those cultured in the control substrate without strain cues were not (Figure 5E and Supplementary Figure S5). We further noted that 8 days of mechanical stimulation induced higher expression of αSMA (Figure 5F) and mRNA levels of ACTA2 and CNN1 (Figure 5G), commonly referred to as αSMA and calponin, respectively. Thus, SMC differentiation from BMSC and their proliferation significantly increased in response to cyclic stretching of the PLCL membrane (Figure 5H), which further indicated that stretch-induced mechanotransduction activated downstream pathways of cell proliferation. These results demonstrate that cyclic stretching enhances MSC differentiation into the SMC, followed by the promotion of cell proliferation.

## 4 Discussions

Biomaterials have been extensively studied in tissue engineering owing to their high demand in medical applications (Kon et al., 2011; Kantak and Bharate, 2021). Scaffolds are polymers, hydrogels, or ceramics, depending on the target application. Among these, polymers have gained attention because of their potential applications in tissue engineering and biomedicine. PLCL has unique properties, such as biodegradability, biocompatibility, controllable mechanical strength, and extreme flexibility (Jeong et al., 2004). Hence, PLCL has been actively investigated for the soft tissue engineering of blood vessels (vasoconstriction and vasodilation), tendons, ligaments, and smooth muscles (J. Lee et al., 2010; Zhu et al., 2018). Thus, we used PLCL as a scaffold and proposed a simple method for fabricating a porosity-controlled PLCL matrix based on the crystallization of copolymer chains in a combination of solvents and non-solvents. In this case, crystallization-related morphological control is primarily dominated by other factors such as the solvent, molecular weight, and polymer ratio. We used solvents with or without different non-solvents to control the morphology or porosity of PLCL by varying the evaporation rate.

The porosity of scaffolds plays a critical role in determining their applications and functions. Porous scaffolds can be used as supports for three-dimensional tissue regeneration, gas or drug storage, and filters (D. Wu et al., 2012). We demonstrated that an increased ratio of non-solvents resulted in increased chain aggregation of PLCL, which led to lower porosity and improved mechanical properties. Solvent polarity is related to the capacity to solvate other charged polar substances (Katritzky et al., 2004). Thus, solvent polarity plays a critical role in solubility (i.e., the solubility of a polar solute in a polar solvent). Among the solvents tested, MeOH/DW was the most polar solvent relative to MeOH and MeOH/CF., whereas the rapid evaporation of CF induced an unstable jet, which led to an irreproducible PLCL matrix. These factors determine the morphology and mechanical properties of the PLCL matrices.

Increasing evidence suggests that biophysical cues can improve MSC differentiation (Kim S. Y. et al., 2015; Andalib et al., 2016). One well-known method for this application is controlling the stiffness of the cell substrate because lineage-specific microenvironment stiffness drives MSC differentiation (D. A. Lee et al., 2011). Another promising biophysical cue for inducing MSC differentiation is the application of mechanical forces. For MSC differentiation, we used a PLCL matrix crystallized with IPA/DW, which showed the lowest porosity and mechanical properties.

Our system offers a notable advantage in terms of cost-effectiveness compared with the previously mentioned studies. Traditional approaches have often utilized differentiation media containing relatively expensive components such as growth factors (J. S. Park et al., 2011; Vater et al., 2011). Although some groups have employed stretched models to induce mechanical strain (Jiang et al., 2016; J; Wu et al., 2018), such systems require expensive electrical equipment. In contrast, our study introduced an inexpensive, custom-made strain system, demonstrating a cost-efficient approach. Using an effective mechanical strain application setup, we demonstrated the differentiation of MSCs into SMCs in standard culture media. Thus, this approach not only reduces the financial burden associated with specialized media but also eliminates the need for expensive stretching devices. Cyclic stretching was repeated on the MSCs for 8 days using a custom-made bioreactor system. Owing to the limited size of the membrane where cells are exposed to mechanical strain, high-throughput and large-scale industrial applications remain challenging. In line with previous studies, our results demonstrated that cyclic stretching induces greater differentiation into SMCs and higher proliferation of MSCs (Ghazanfari et al., 2009; Morioka et al., 2011), which further supports the use of our bioreactor system for stem cell-based vessel regeneration.

## 5 Conclusion

We demonstrated a robust method for fabricating a porosity-controlled PLCL membrane using a combination of solvents and non-solvents. We confirmed that the increased chain aggregation of PLCL using IPA/DW led to a mechanically stable PLCL membrane with nonporous morphology. To mimic a highly dynamic vascular wall exposed to continuous compression and expansion, we designed a PLCL membrane-equipped microfluidic device that could fine-tune the mechanical stimuli applied to the cells. Our custom-made microfluidic bioreactor system can recapitulate stretch-induced stem cell differentiation into SMCs. This study provides a critical design concept for optimizing the microstructure of polymeric scaffolds for tissue regeneration.

## Data Availability

The original contributions presented in the study are included in the article/Supplementary Material, further inquiries can be directed to the corresponding authors.

## References

[B1] Altman G. H. Stark P. Martin I. Richmond J. C. Vunjak-Novakovic G. Lu H. H. (2002). Advanced bioreactor with controlled application of multi-dimensional strain for tissue engineering. J. biomechanical Eng. 124 (6), 742–749. 10.1115/1.1519280 12596643

[B2] Andalib M. N. Dzenis Y. Donahue H. J. Lim J. Y. (2016). Biomimetic substrate control of cellular mechanotransduction. Biomater. Res. 20, 11. 10.1186/s40824-016-0059-1 27134756 PMC4850706

[B3] Chamberlain G. Fox J. Ashton B. Middleton J. (2007). Concise review: mesenchymal stem cells: their phenotype, differentiation capacity, immunological features, and potential for homing. Stem Cells 25 (11), 2739–2749. 10.1634/stemcells.2007-0197 17656645

[B4] Choi K.-M. Seo Y.-K. Yoon H.-H. Song K.-Y. Kwon S.-Y. Lee H.-S. (2007). Effects of mechanical stimulation on the proliferation of bone marrow-derived human mesenchymal stem cells. Biotechnol. Bioprocess Eng. 12, 601–609. 10.1007/bf02931075

[B5] Chowdhury F. Na S. Li D. Poh Y.-C. Tanaka T. S. Wang F. (2010). Material properties of the cell dictate stress-induced spreading and differentiation in embryonic stem cells. Nat. Mater. 9 (1), 82–88. 10.1038/nmat2563 19838182 PMC2833279

[B6] Delaine-Smith R. M. Reilly G. C. (2012). Mesenchymal stem cell responses to mechanical stimuli. Muscles, ligaments tendons J. 2 (3), 169–180. 23738294 PMC3666521

[B7] Diban N. Stamatialis D. F. (2011). Functional polymer scaffolds for blood vessel tissue engineering. United States: Wiley. Paper presented at the Macromolecular Symposia.

[B8] Discher D. E. Janmey P. Wang Y.-l. (2005). Tissue cells feel and respond to the stiffness of their substrate. Science 310 (5751), 1139–1143. 10.1126/science.1116995 16293750

[B9] Engler A. J. Sen S. Sweeney H. L. Discher D. E. (2006). Matrix elasticity directs stem cell lineage specification. Cell 126 (4), 677–689. 10.1016/j.cell.2006.06.044 16923388

[B10] Even-Ram S. Artym V. Yamada K. M. (2006). Matrix control of stem cell fate. Cell 126 (4), 645–647. 10.1016/j.cell.2006.08.008 16923382

[B11] Fernández J. Etxeberria A. Sarasua J. R. (2012). Synthesis, structure and properties of poly (L-lactide-co-ε-caprolactone) statistical copolymers. J. Mech. Behav. Biomed. Mat. 9, 100–112. 10.1016/j.jmbbm.2012.01.003 22498288

[B12] Friedenstein A. Chailakhjan R. Lalykina K. (1970). The development of fibroblast colonies in monolayer cultures of Guinea‐pig bone marrow and spleen cells. Cell Prolif. 3 (4), 393–403. 10.1111/j.1365-2184.1970.tb00347.x 5523063

[B13] Ghazanfari S. Tafazzoli-Shadpour M. Shokrgozar M. A. (2009). Effects of cyclic stretch on proliferation of mesenchymal stem cells and their differentiation to smooth muscle cells. Biochem. biophysical Res. Commun. 388 (3), 601–605. 10.1016/j.bbrc.2009.08.072 19695226

[B14] He W.-N. Xu J.-T. (2012). Crystallization assisted self-assembly of semicrystalline block copolymers. Prog. Polym. Sci. 37 (10), 1350–1400. 10.1016/j.progpolymsci.2012.05.002

[B15] Huycke T. R. Miller B. M. Gill H. K. Nerurkar N. L. Sprinzak D. Mahadevan L. (2019). Genetic and mechanical regulation of intestinal smooth muscle development. Cell 179 (1), 90–105. 10.1016/j.cell.2019.08.041 31539501 PMC6756183

[B16] Jeong S. I. Kim S. H. Kim Y. H. Jung Y. Kwon J. H. Kim B.-S. (2004). Manufacture of elastic biodegradable PLCL scaffolds for mechano-active vascular tissue engineering. J. Biomaterials Sci. Polym. Ed. 15 (5), 645–660. 10.1163/156856204323046906 15264665

[B17] Jiang Y. Wang Y. Tang G. (2016). Cyclic tensile strain promotes the osteogenic differentiation of a bone marrow stromal cell and vascular endothelial cell co-culture system. Archives Biochem. biophysics 607, 37–43. 10.1016/j.abb.2016.08.015 27562627

[B18] Kantak M. N. Bharate S. S. (2021). Analysis of clinical trials on biomaterial and therapeutic applications of chitosan: a review. Carbohydr. Polym. 278, 118999. 10.1016/j.carbpol.2021.118999 34973801

[B19] Katritzky A. R. Fara D. C. Yang H. Tämm K. Tamm T. Karelson M. (2004). Quantitative measures of solvent polarity. Chem. Rev. 104 (1), 175–198. 10.1021/cr020750m 14719974

[B20] Kim S. H. Kim S. H. Jung Y. (2015a). TGF-β3 encapsulated PLCL scaffold by a supercritical CO2-HFIP co-solvent system for cartilage tissue engineering. J. Control. Release 206, 101–107. 10.1016/j.jconrel.2015.03.026 25804870

[B21] Kim S. Y. Kang J. H. Seo W. S. Lee S. W. Oh N. S. Cho H. K. (2015b). Effect of topographical control by a micro-molding process on the activity of human Mesenchymal Stem Cells on alumina ceramics. Biomater. Res. 19, 23. 10.1186/s40824-015-0045-z 26543592 PMC4634586

[B22] Kon E. Delcogliano M. Filardo G. Busacca M. Di Martino A. Marcacci M. (2011). Novel nano-composite multilayered biomaterial for osteochondral regeneration: a pilot clinical trial. Am. J. sports Med. 39 (6), 1180–1190. 10.1177/0363546510392711 21310939

[B23] Krampera M. Pizzolo G. Aprili G. Franchini M. (2006). Mesenchymal stem cells for bone, cartilage, tendon and skeletal muscle repair. Bone 39 (4), 678–683. 10.1016/j.bone.2006.04.020 16765663

[B24] Kunzler T. P. Drobek T. Schuler M. Spencer N. D. (2007). Systematic study of osteoblast and fibroblast response to roughness by means of surface-morphology gradients. Biomaterials 28 (13), 2175–2182. 10.1016/j.biomaterials.2007.01.019 17275082

[B25] Lee D. A. Knight M. M. Campbell J. J. Bader D. L. (2011). Stem cell mechanobiology. J. Cell. Biochem. 112 (1), 1–9. 10.1002/jcb.22758 20626029

[B26] Lee G. Lee J. Kim J. Choi H. S. Kim J. Lee S. (2017a). Single microfluidic electrochemical sensor system for simultaneous multi-pulmonary hypertension biomarker analyses. Sci. Rep. 7 (1), 7545–7548. 10.1038/s41598-017-06144-9 28790334 PMC5548735

[B27] Lee G. H. Lee J. S. Lee G.-H. Joung W. Y. Kim S. H. Lee S. H. (2017b). Networked concave microwell arrays for constructing 3D cell spheroids. Biofabrication 10 (1), 015001. 10.1088/1758-5090/aa9876 29190216

[B28] Lee J. Guarino V. Gloria A. Ambrosio L. Tae G. Kim Y. H. (2010). Regeneration of Achilles' tendon: the role of dynamic stimulation for enhanced cell proliferation and mechanical properties. J. Biomaterials Sci. Polym. Ed. 21 (8-9), 1173–1190. 10.1163/092050609X12471222313524 20507714

[B29] Maul T. M. Chew D. W. Nieponice A. Vorp D. A. (2011). Mechanical stimuli differentially control stem cell behavior: morphology, proliferation, and differentiation. Biomechanics Model. Mechanobiol. 10 (6), 939–953. 10.1007/s10237-010-0285-8 PMC3208754 21253809

[B30] Meng X. Leslie P. Zhang Y. Dong J. (2014). Stem cells in a three-dimensional scaffold environment. SpringerPlus 3 (1), 80. 10.1186/2193-1801-3-80 24570851 PMC3931863

[B31] Morioka M. Parameswaran H. Naruse K. Kondo M. Sokabe M. Hasegawa Y. (2011). Microtubule dynamics regulate cyclic stretch-induced cell alignment in human airway smooth muscle cells. PloS one 6 (10), e26384. 10.1371/journal.pone.0026384 22022610 PMC3195692

[B32] Mun C. H. Jung Y. Kim S. H. Kim H. C. Kim S. H. (2013). Effects of pulsatile bioreactor culture on vascular smooth muscle cells seeded on electrospun poly (lactide‐co‐ε‐caprolactone) scaffold. Artif. organs 37 (12), E168–E178. 10.1111/aor.12108 23834728

[B33] Mun C. H. Jung Y. Kim S.-H. Lee S.-H. Kim H. C. Kwon I. K. (2012). Three-dimensional electrospun poly (lactide-Co-ɛ-Caprolactone) for small-diameter vascular grafts. Tissue Eng. Part A 18 (15-16), 1608–1616. 10.1089/ten.TEA.2011.0695 22462723

[B34] Obradovic B. Radisic M. Vunjak-Novakovic G. (2010). Biomimetic approaches to design of tissue engineering bioreactors *Advances in regenerative medicine: Role of nanotechnology, and engineering principles* . Springer, 115–129.

[B35] Oh S. H. Kim T. H. Im G. I. Lee J. H. J. B. (2010). Investigation of pore size effect on chondrogenic differentiation of adipose stem cells using a pore size gradient scaffold. Biomacromolecules 11 (8), 1948–1955. 10.1021/bm100199m 20690707

[B36] Osol G. (1995). Mechanotransduction by vascular smooth muscle. J. Vasc. Res. 32 (5), 275–292. 10.1159/000159102 7578796

[B37] Park J. S. Chu J. S. Tsou A. D. Diop R. Tang Z. Wang A. (2011). The effect of matrix stiffness on the differentiation of mesenchymal stem cells in response to TGF-β. Biomaterials 32 (16), 3921–3930. 10.1016/j.biomaterials.2011.02.019 21397942 PMC3073995

[B38] Park J. Y. Hwang C. M. Lee S. H. Lee S.-H. (2007). Gradient generation by an osmotic pump and the behavior of human mesenchymal stem cells under the fetal bovine serum concentration gradient. Lab a Chip 7 (12), 1673–1680. 10.1039/b710777c 18030386

[B39] Phillip W. A. Hillmyer M. A. Cussler E. J. M. (2010). Cylinder orientation mechanism in block copolymer thin films upon solvent evaporation. Macromolecules 43 (18), 7763–7770. 10.1021/ma1012946

[B40] Potter C. M. Lao K. H. Zeng L. Xu Q. (2014). Role of biomechanical forces in stem cell vascular lineage differentiation. Arteriosclerosis, thrombosis, Vasc. Biol. 34 (10), 2184–2190. 10.1161/ATVBAHA.114.303423 25012135

[B41] Rammensee S. Kang M. S. Georgiou K. Kumar S. Schaffer D. V. J. S. c. (2017). Dynamics of mechanosensitive neural stem cell differentiation. Dyn. mechanosensitive neural stem Cell Differ. 35 (2), 497–506. 10.1002/stem.2489 PMC5285406 27573749

[B42] Razali M. Didaskalou C. Kim J. F. Babaei M. Drioli E. Lee Y. M. (2017). Exploring and exploiting the effect of solvent treatment in membrane separations. ACS Appl. Mat. Interfaces 9 (12), 11279–11289. 10.1021/acsami.7b01879 28276673

[B43] Richardson S. M. Curran J. M. Chen R. Vaughan-Thomas A. Hunt J. A. Freemont A. J. (2006). The differentiation of bone marrow mesenchymal stem cells into chondrocyte-like cells on poly-L-lactic acid (PLLA) scaffolds. Biomaterials 27 (22), 4069–4078. 10.1016/j.biomaterials.2006.03.017 16569429

[B44] Sachlos E. Czernuszka J. (2003). Making tissue engineering scaffolds work. Review: the application of solid freeform fabrication technology to the production of tissue engineering scaffolds. Eur. Cell Mater 5 (29), 29–39. 10.22203/ecm.v005a03 14562270

[B45] Saha S. K. Tsuji H. J. R. Polymers F. (2006). Effects of rapid crystallization on hydrolytic degradation and mechanical properties of poly (l-lactide-co-ε-caprolactone). React. Funct. Polym. 66 (11), 1362–1372. 10.1016/j.reactfunctpolym.2006.03.020

[B46] Sarasua J.-R. Prud'Homme R. E. Wisniewski M. Le Borgne A. Spassky N. J. M. (1998). Crystallization and melting behavior of polylactides. Cryst. melting Behav. polylactides 31 (12), 3895–3905. 10.1021/ma971545p

[B47] Shaikh F. M. O'Brien T. P. Callanan A. Kavanagh E. G. Burke P. E. Grace P. A. (2010). New pulsatile hydrostatic pressure bioreactor for vascular tissue‐engineered constructs. Artif. organs 34 (2), 153–158. 10.1111/j.1525-1594.2009.00766.x 19995361

[B48] Shin Y. M. Kim K.-S. Lim Y. M. Nho Y. C. Shin H. (2008). Modulation of spreading, proliferation, and differentiation of human mesenchymal stem cells on gelatin-immobilized poly (l-lactide-co-ϵ-caprolactone) substrates. Biomacromolecules 9 (7), 1772–1781. 10.1021/bm701410g 18558737

[B49] Sodian R. Lemke T. Fritsche C. Hoerstrup S. P. Fu P. Potapov E. V. (2002). Tissue-engineering bioreactors: a new combined cell-seeding and perfusion system for vascular tissue engineering. Tissue Eng. 8 (5), 863–870. 10.1089/10763270260424222 12459065

[B50] Sun Y. Chen C. S. Fu J. (2012). Forcing stem cells to behave: a biophysical perspective of the cellular microenvironment. Annu. Rev. biophysics 41, 519–542. 10.1146/annurev-biophys-042910-155306 PMC4123632 22404680

[B51] Sun Y. Wan B. Wang R. Zhang B. Luo P. Wang D. (2022). Mechanical stimulation on mesenchymal stem cells and surrounding microenvironments in bone regeneration: regulations and applications. Front. Cell Dev. Biol. 10, 2. 10.3389/fcell.2022.808303 PMC8815029 35127684

[B52] Teixeira G. Q. Barrias C. C. Lourenço A. F. Goncalves R. (2014). A multi-compartment holder for spinner flasks improves expansion and osteogenic differentiation of mesenchymal stem cells in 3D scaffolds. Tissue Eng. 20(12):984–993. 10.1089/ten.TEC.2014.0067 PMC4241866 24650268

[B53] Tuan R. S. Boland G. Tuli R. (2002). Adult mesenchymal stem cells and cell-based tissue engineering. Arthritis Res. Ther. 5 (1), 32–45. 10.1186/ar614 12716446 PMC154434

[B54] Vater C. Kasten P. Stiehler M. (2011). Culture media for the differentiation of mesenchymal stromal cells. Acta biomater. 7 (2), 463–477. 10.1016/j.actbio.2010.07.037 20688199

[B55] Wang J. H. C. Thampatty B. P. (2008). Mechanobiology of adult and stem cells. Int. Rev. Cell Mol. Biol. 271, 301–346. 10.1016/S1937-6448(08)01207-0 19081546

[B56] Watt F. M. Hogan B. (2000). Out of Eden: stem cells and their niches. Science 287 (5457), 1427–1430. 10.1126/science.287.5457.1427 10688781

[B57] Webb R. C. (2003). Smooth muscle contraction and relaxation. Adv. physiology Educ. 27 (4), 201–206. 10.1152/advan.00025.2003 14627618

[B58] Winer J. P. Janmey P. A. McCormick M. E. Funaki M. (2008). Bone marrow-derived human mesenchymal stem cells become quiescent on soft substrates but remain responsive to chemical or mechanical stimuli. Tissue Eng. Part A 15 (1), 147–154. 10.1089/ten.tea.2007.0388 18673086

[B59] Wu D. Xu F. Sun B. Fu R. He H. Matyjaszewski K. (2012). Design and preparation of porous polymers. Chem. Rev. 112 (7), 3959–4015. 10.1021/cr200440z 22594539

[B60] Wu J. Zhao J. Sun L. Pan Y. Wang H. Zhang W.-B. (2018). Long non-coding RNA H19 mediates mechanical tension-induced osteogenesis of bone marrow mesenchymal stem cells via FAK by sponging miR-138. Bone 108, 62–70. 10.1016/j.bone.2017.12.013 29253550

[B61] Yan L. Chouw N. Huang L. Kasal B. J. C. Materials B. (2016). Effect of alkali treatment on microstructure and mechanical properties of coir fibres, coir fibre reinforced-polymer composites and reinforced-cementitious composites. Constr. Build. Mat. 112, 168–182. 10.1016/j.conbuildmat.2016.02.182

[B62] Zhang S. Chen L. Liu T. Wang Z. Wang Y. (2014). Integration of single-layer skin hollow fibers and scaffolds develops a three-dimensional hybrid bioreactor for bioartificial livers. J. Mater. Sci. Mater. Med. 25 (1), 207–216. 10.1007/s10856-013-5033-z 23963686

[B63] Zhu M. Wu Y. Li W. Dong X. Chang H. Wang K. (2018). Biodegradable and elastomeric vascular grafts enable vascular remodeling. Biomaterials 183, 306–318. 10.1016/j.biomaterials.2018.08.063 30189358

