# Supplementary material for: Stretching of porous poly (l-lactide-co-ε-caprolactone) membranes regulates the differentiation of mesenchymal stem cells
Source: Front Cell Dev Biol. 2024 Jan 25;12:1303688. doi: 10.3389/fcell.2024.1303688 (PMC10850303; doi:10.3389/fcell.2024.1303688)
Supplement: Supplementary file 2 [file DataSheet1.docx]

**Stretching of porous poly (l-lactide-co-ε-caprolactone) membranes regulates the differentiation of mesenchymal stem cells**

**Geonhui Lee^1,2^, Seong-Beom Han^3^, Soo Hyun Kim^3,4^, Sangmoo Jeong^1,2*^, and Dong-Hwee Kim^3,4,5*^**

^1^ Department of Chemical and Biomolecular Engineering, Johns Hopkins University, Baltimore, MD, USA

^2^ Institute for NanoBioTechnology, Johns Hopkins University, Baltimore, MD, USA

^3^ KU-KIST Graduate School of Converging Science and Technology, Korea University, Seoul, Republic of Korea

^4^ Biomaterials Research Center, Biomedical Research Division, Korea Institute of Science and Technology, Seoul, Republic of Korea

^5^ Department of Integrative Energy Engineering, College of Engineering, Korea University, Seoul, Republic of Korea

*** Correspondence:**

Dong-Hwee Kim ([donghweekim@korea.ac.kr](mailto:donghweekim@korea.ac.kr)) or Sangmoo Jeong ([sjeong@jhu.edu](mailto:sjeong@jhu.edu))

**Keywords: mechanotransduction, PLCL, bioreactor, mesenchymal stem cell, differentiation, smooth muscle cell**

**Supplementary data**


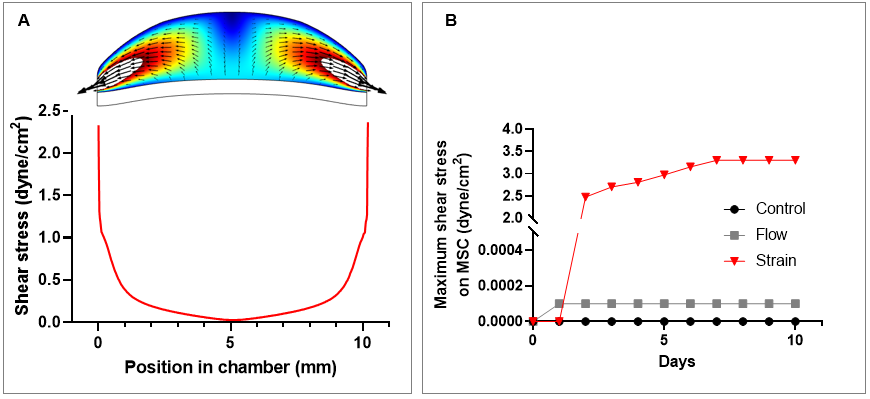


**Fig. S1. Simulation of shear stress applied to the cell in the microfluidic device.**

(A) Distribution of shear stress crossing the chamber in case of the maximum stretch of PLCL membrane. (B) Comparison of the maximum shear stress of the cells with or without flow or strain on the membrane.


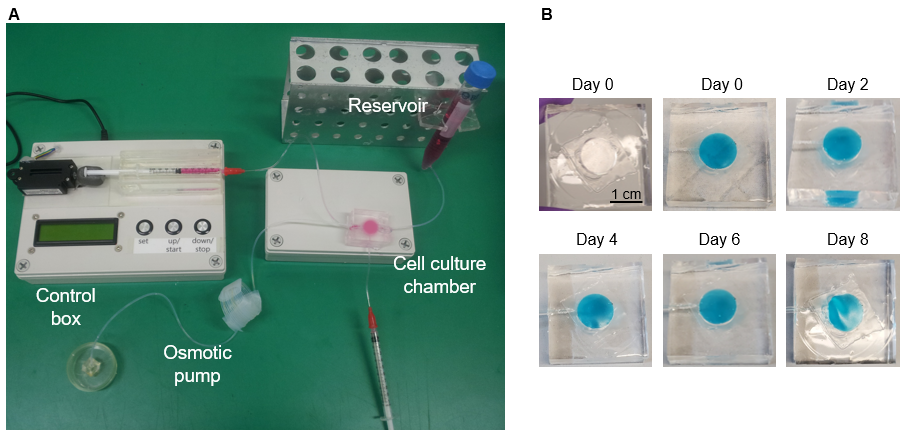


**Fig. S2. Image of microfluidic device.** (A) Photo of microfluidic device connected to bioreactor and media reservoir to generate stretch and shear stress. (B) Photographs depicting the time-dependent stretching of the PLCL matrix. No leakage observed through the PLCL matrix crystallized by MeOH/DW through 8 days of stretching**.**


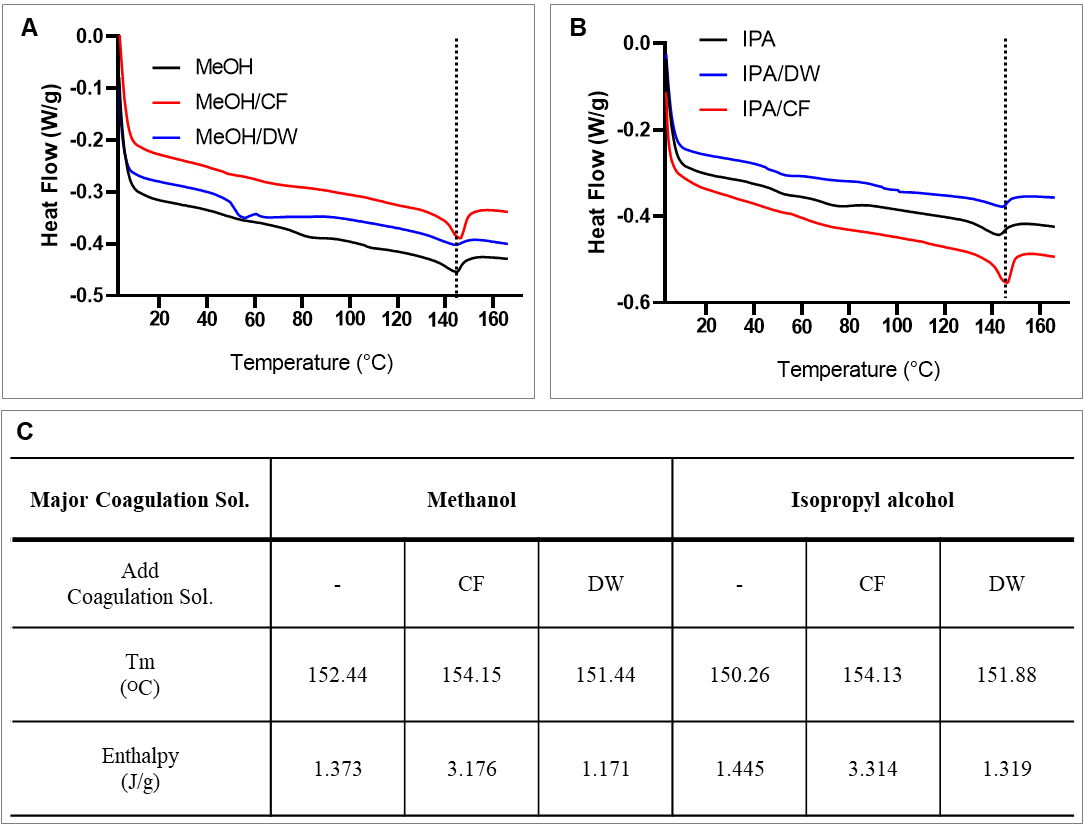


**Fig. S3. DSC analysis of the PLCL membrane crystallized with diverse solvents.** Heat flow of PLCL membrane crystalized with MeOH- (A) and IPA- (B) based solvents. The value of melting temperature (Tm) and melting enthalpy (J/g) are reported.


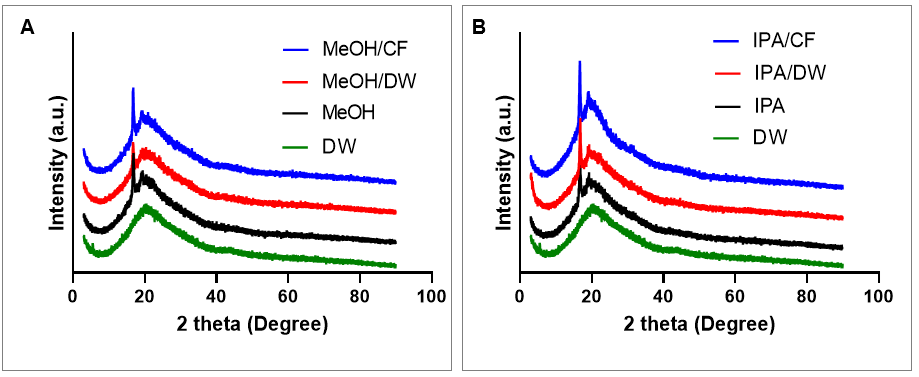


**Fig. S4. WAXD analysis of the PLCL membrane crystalized with diverse solvents.**


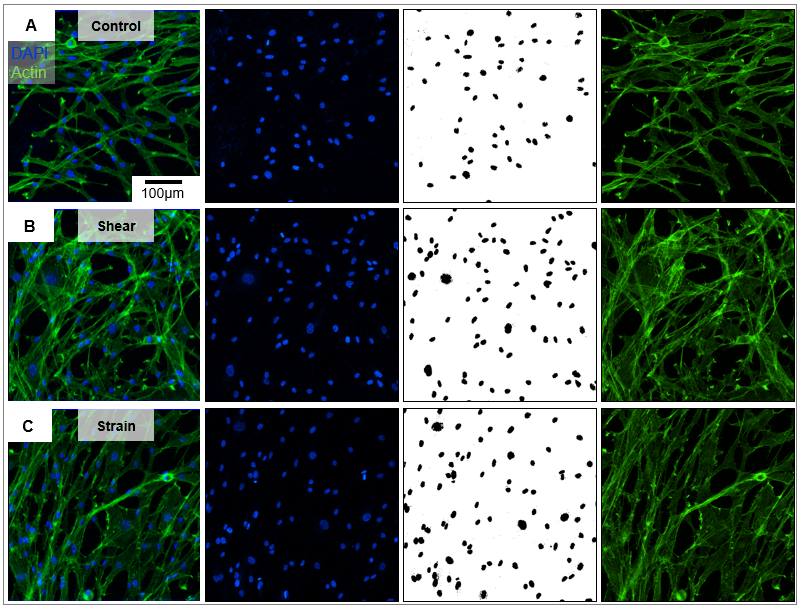


**Fig. S5. Cell alignment of BMSC in control (A), flow (B), and strain (C) conditions of the PLCL membranes.** Actin filaments were visualized by phalloidin (green) in DAPI (blue)-stained BMSCs.
